# Gut Microbiota and Metabolome Alterations Associated with Parkinson’s Disease

**DOI:** 10.1128/mSystems.00561-20

**Published:** 2020-09-15

**Authors:** Sarah Vascellari, Vanessa Palmas, Marta Melis, Silvia Pisanu, Roberto Cusano, Paolo Uva, Daniela Perra, Veronica Madau, Marianna Sarchioto, Valentina Oppo, Nicola Simola, Micaela Morelli, Maria Laura Santoru, Luigi Atzori, Maurizio Melis, Giovanni Cossu, Aldo Manzin

**Affiliations:** a Department of Biomedical Sciences, Section of Microbiology and Virology, University of Cagliari, Cagliari, Italy; b Neurology Service and Stroke Unit, AO Brotzu Hospital, Cagliari, Italy; c CRS4, Science and Technology Park Polaris, Piscina Manna, Pula, Cagliari, Italy; d Department of Medical Sciences and Public Health, University of Cagliari, Cagliari, Italy; e Department of Biomedical Sciences, Section of Neuropsychopharmacology, University of Cagliari, Cagliari, Italy; f Department of Biomedical Sciences, Oncology and Molecular Pathology Unit, University of Cagliari, Cagliari, Italy; University of Massachusetts Medical School

**Keywords:** 16S RNA, gut microbiota, PD, metabolome

## Abstract

Parkinson’s disease is a neurodegenerative disorder characterized by the accumulation of intracellular aggregates of misfolded alpha-synuclein along the cerebral axis. Several studies report the association between intestinal dysbiosis and Parkinson’s disease, although a cause-effect relationship remains to be established. Herein, the gut microbiota composition of 64 Italian patients with Parkinson’s disease and 51 controls was determined using a next-generation sequencing approach. A real metagenomics shape based on gas chromatography-mass spectrometry was also investigated. The most significant changes within the Parkinson’s disease group highlighted a reduction in bacterial taxa, which are linked to anti-inflammatory/neuroprotective effects, particularly in the *Lachnospiraceae* family and key members, such as *Butyrivibrio, Pseudobutyrivibrio, Coprococcus*, and *Blautia*. The direct evaluation of fecal metabolites revealed changes in several classes of metabolites. Changes were seen in lipids (linoleic acid, oleic acid, succinic acid, and sebacic acid), vitamins (pantothenic acid and nicotinic acid), amino acids (isoleucine, leucine, phenylalanine, glutamic acid, and pyroglutamic acid) and other organic compounds (cadaverine, ethanolamine, and hydroxy propionic acid). Most modified metabolites strongly correlated with the abundance of members belonging to the *Lachnospiraceae* family, suggesting that these gut bacteria correlate with altered metabolism rates in Parkinson’s disease.

**IMPORTANCE** To our knowledge, this is one of the few studies thus far that correlates the composition of the gut microbiota with the direct analysis of fecal metabolites in patients with Parkinson’s disease. Overall, our data highlight microbiota modifications correlated with numerous fecal metabolites. This suggests that Parkinson’s disease is associated with gut dysregulation that involves a synergistic relationship between gut microbes and several bacterial metabolites favoring altered homeostasis. Interestingly, a reduction of short-chain fatty acid (SCFA)-producing bacteria influenced the shape of the metabolomics profile, affecting several metabolites with potential protective effects in the Parkinson group. On the other hand, the extensive impact that intestinal dysbiosis has at the level of numerous metabolic pathways could encourage the identification of specific biomarkers for the diagnosis and treatment of Parkinson’s disease, also in light of the effect that specific drugs have on the composition of the intestinal microbiota.

## Abstract (precis)

To our knowledge, this is one of the few studies thus far that correlates the composition of the gut microbiota with the direct analysis of fecal metabolites in patients with Parkinson’s disease. Overall, our data highlight microbiota modifications correlated with numerous fecal metabolites. This suggests that Parkinson’s disease is associated with gut dysregulation that involves a synergistic relationship between gut microbes and several bacterial metabolites favoring altered homeostasis. Interestingly, a reduction of short-chain fatty acid (SCFA)-producing bacteria influenced the shape of the metabolomics profile, affecting several metabolites with potential protective effects in the Parkinson group. On the other hand, the extensive impact that intestinal dysbiosis has at the level of numerous metabolic pathways could encourage the identification of specific biomarkers for the diagnosis and treatment of Parkinson’s disease, also in light of the effect that specific drugs have on the composition of the intestinal microbiota.

## INTRODUCTION

Parkinson’s disease (PD) is a neurodegenerative disorder characterized by the intracellular accumulation of α-synuclein (α-syn) aggregates (Lewy bodies) at various levels of the cerebral axis, including the central nervous system (CNS) and the enteric nervous system (ENS) (1). Clinical and neuropathological evidence indicates that the neurodegenerative changes in PD are accompanied by gastrointestinal (GI) dysregulation; however, whether this precedes or follows motor impairment remains to be established (2). Constipation is the most common premotor symptom in PD; it can affect more than 70% of PD patients (3) and can promote pathogenesis more than 10 years before the onset of clinical symptoms (2). Nevertheless, accumulation and aggregation of misfolded α-syn in the gut, coupled with neurodegeneration in the ENS (4), seems to start up to 20 years before the onset of neurodegeneration in the CNS (5), thus supporting the “gut-to-brain” hypothesis (6). Interactions between the intestine and brain are known to be modulated by the intestinal microbiota through immunological, neuroendocrine, and direct neurochemical mechanisms (7). Gut bacteria and bacterial metabolites could play a role in the pathogenesis of PD by promoting a proinflammatory environment in the gut (8, 9). Furthermore, intestinal inflammation associated with dysbiosis may contribute to the misfolding of α-syn (10, 11).

Recently, different studies have described gut microbial alterations associated with PD patients. The results are somewhat heterogeneous, and there is no explicit agreement about which bacteria community might be involved (12–20).

Moreover, gut microbiota modifications could reflect changes in bacterial metabolites. To date, several studies have investigated the metabolomics profile of PD patients in different biological samples, such as cerebrospinal fluid, blood, and urine (21–26). However, only a few studies have explored the fecal metabolome that mirrors the status of colonic bacteria and also links the symbiotic microbiota and health. A better understanding of the microbiota and metabolomics profile in fecal samples could elucidate the interactions between host/bacterial metabolisms, gut microbes, and disease. In turn, this knowledge may provide critical information for the implementation of better diet in PD.

Therefore, this study aimed to investigate the composition and structure of the fecal microbiota in a cohort of Italian PD patients (PDs) compared to healthy subjects (HCs) using 16S rRNA gene sequencing. Furthermore, intending to understand the functional contribution of the microbial community, we used direct analysis of the fecal metabolome to identify potential metabolic alterations associated with the microbiota in PD patients.

## RESULTS

### Patients with Parkinson’s disease display altered microbiota composition compared to healthy subjects.

**FIG 1:**
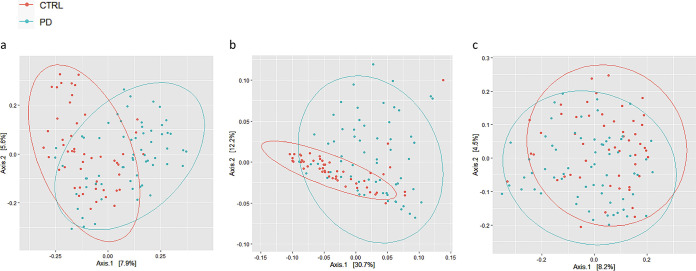
Beta-diversity analysis was presented as a two-dimensional (2D) plot based on principal coordinate analysis (PCoA). The statistical significance was assessed using permutational multivariate analysis of variance (PERMANOVA). (a) Bray Curtis (*P* = 0.001; *F* = 4.217; R2 = 0.036); (b) UniFrac weighted (*P* = 0.001; *F* = 9.628; R2 = 0.079); (c) UniFrac unweighted (*P* = 0.001; *F* = 3.255; R2  =  0.028). CTRL, control.

**FIG 2:**
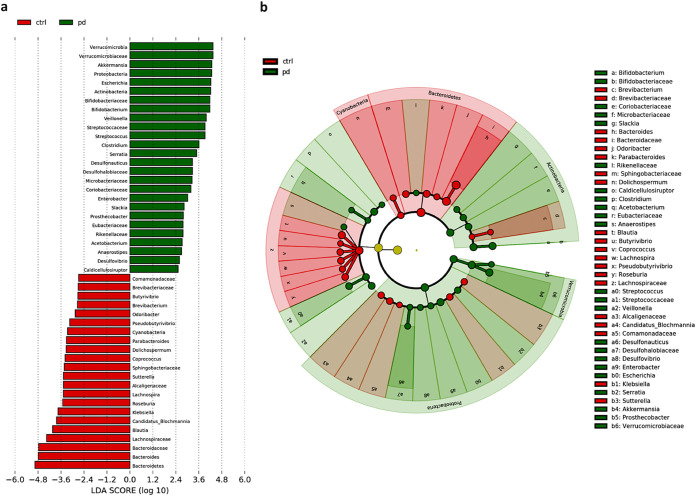
LEfSE analysis. The plot was generated using a Galaxy computational tool. (a) The bar plots represent the significantly differential taxa between PD patients (green) and controls (ctrl) (red), based on effect size (LDA score [log 10] > 2). Enriched taxa in PD patients (positive LDA score) and enriched taxa in controls (negative LDA score). Differences among classes were obtained by the Kruskal-Wallis test (α = 0.05). (b) Cladogram showed the differences in enriched taxa in PD (green) versus enriched taxa in controls (red). Differences among classes were obtained by the Kruskal-Wallis test (α = 0.05).

Characteristics of the 64 patients with PD and 51 HCs are shown in Table 1. The bacterial communities in the PD patients and controls were analyzed at different taxonomic levels. *Firmicutes*, *Bacteroidetes*, *Proteobacteria*, *Actinobacteria*, and *Verrucomicrobia* were the five most abundant phyla, and altogether, they comprised more than 96% of all sequences. We detected no significant differences between cases and controls in alpha-diversity (species richness of a group) indexes (*P* > 0.05) (data not shown). We also assessed potential community-level differences between samples using beta-diversity analysis, and the weighted and unweighted dissimilarity significantly differed between cases and controls (R2 = 0.079, *P* < 0.001, weighted; R2 = 0.028, *P* < 0.001, unweighted) (Fig. 1b and c). The Mann-Whitney U test followed by the Benjamini and Hochberg false discovery rate (FDR) correction test for multiple comparisons was implemented to identify the different taxa within the groups. The FDR-corrected significant differences were also plotted using linear discriminant analysis (LDA) effect size (LEfSe) method (Fig. 2). The relative abundance of each taxon in the two study groups is reported in Table 2. *Actinobacteria*, *Proteobacteria*, and *Verrucomicrobia* were significantly enriched in diseased subjects, while *Bacteroidetes* and *Cyanobacteria* were significantly decreased; also, members of the *Firmicutes* were decreased; however, there was no significant difference in distribution between the PD and HC groups (*P* > 0.05).

**TABLE 1:** Subject characteristics^*a*^

Variable	PD patients (*n* = 64)	Control subjects (*n* = 51)
Age (yr), mean ± SD	71.39 + 10.99	51.67 + 12.42
BMI, mean ± SD	26.07 + 4.18	23.70 + 3.46
Sex, *n* (%)		
Male	44 (68.75)	31 (60.78)
Female	20 (31.25)	20 (39.22)
Constipation, *n* (%)	37 (57.81)	0 (0)
Coffee consumption, *n* (%)		
Yes	40 (62.50)	44 (86.27)
No	24 (37.50)	7 (13.73)
Smoking status, *n* (%)		
Yes	7 (10.94)	18 (35.29)
No	57 (89.06)	33 (64.71)
Phenotype, *n* (%)		
Tremor-dominant	22 (34.37)	
Rigid-akinetic	26 (40.63)	
Dyskinetic	16 (25.00)	
Treatment, *n* (%)		
l-DOPA	64 (100)	

a PD, Parkinson’s disease; *n*, case number; SD, standard deviation; BMI, body mass index; l-DOPA, l-3,4-dihydroxyphenylalanine.

**TABLE 2:** Statistically significant differences in bacterial taxa between PD patients versus healthy controls^*a*^

Phylum	Family	Genus	↓/↑^*b*^	MD^*c*^	PD (%)^*d*^	HC (%)^*d*^	*P* value	FDR- adjusted *P* value^*e*^
*Firmicutes*	*Thermoanaerobacterales* Incertae sedis	*Caldicellulosiruptor*	↑	0.226	0.121	0.058	0.010	0.014
	*Veillonellaceae*	*Veillonella*	↑	0.599	1.994	0.277	0.000	0.000
	*Clostridiaceae*	*Clostridium*	↑	0.169	3.133	2.154	0.012	0.016
	*Streptococcaceae*		↑	0.526	1.737	0.155	0.000	0.000
		*Streptococcus*	↑	0.513	1.730	0.153	0.000	0.000
	*Eubacteriaceae*		↑	0.287	0.120	0.062	0.000	0.000
		*Acetobacterium*	↑	0.273	0.115	0.059	0.000	0.000
	*Lachnospiraceae*		↓	−0.253	8.000	12.460	0.001	0.002
		*Anaerostipes*	↑	−0.406	0.236	0.144	0.027	0.030
		*Blautia*	↓	−0.243	3.778	5.928	0.001	0.002
		*Lachnospira*	↓	−0.444	0.535	1.086	0.000	0.000
		*Butyrivibrio*	↓	−0.655	0.021	0.113	0.000	0.000
		*Roseburia*	↓	−0.668	1.066	1.777	0.000	0.000
		*Pseudobutyrivibrio*	↓	−0.562	0.160	0.444	0.000	0.000
		*Coprococcus*	↓	−0.409	0.522	0.954	0.003	0.005

*Bacteroidetes*			↓	−0.269	28.546	47.455	0.000	0.000
	*Bacteroidaceae*		↓	−0.297	17.454	29.910	0.000	0.000
		*Bacteroides*	↓	−0.297	17.454	30.383	0.000	0.000
	*Odoribacteriaceae*	*Odoribacter*	↓	−0.432	0.191	0.331	0.001	0.002
	*Porphyromonadaceae*	*Parabacteroides*	↓	−0.245	1.858	2.269	0.046	0.050
	*Rikenellaceae*		↑	1.460	0.112	0.000	0.000	0.000
	*Sphingobacteriaceae*		↓	−0.177	1.045	1.471	0.044	0.049

*Proteobacteria*			↑	0.177	10.548	6.542	0.020	0.023
	*Desulfovibrionaceae*	*Desulfovibrio*	↑	0.278	0.293	0.245	0.020	0.023
	*Desulfohalobiaceae*		↑	0.355	0.185	0.123	0.002	0.003
		*Desulfonauticus*	↑	0.304	0.184	0.122	0.002	0.003
	*Sutterellaceae*	*Sutterella*	↓	−0.643	0.258	0.819	0.000	0.000
	*Alcaligenaceae*		↓	−0.642	0.270	0.837	0.000	0.000
	*Comamonadaceae*		↓	−0.440	0.045	0.127	0.001	0.002
	*Enterobacteriaceae*	*Enterobacter*	↑	0.559	0.404	0.173	0.001	0.002
		*Escherichia*	↑	1.003	3.737	0.259	0.000	0.000
		*Serratia*	↑	1.011	0.672	0.050	0.000	0.000
		*Klebsiella*	↓	−0.723	0.668	0.961	0.000	0.000
		“*Candidatus* Blochmannia”	↓	−1.673	0.042	1.392	0.000	0.000

*Actinobacteria*			↑	0.313	5.524	2.263	0.002	0.003
	*Bifidobacteriaceae*		↑	0.362	4.191	1.289	0.014	0.018
		*Bifidobacterium*	↑	0.356	4.173	1.288	0.016	0.020
	*Coriobacteriaceae*		↑	0.240	0.944	0.562	0.012	0.016
		*Slackia*	↑	0.309	0.309	0.158	0.001	0.002
	*Microbacteriaceae*		↑	0.441	0.677	0.333	0.000	0.000
	*Brevibacteriaceae*		↓	0.288	0.072	0.121	0.000	0.000
		*Brevibacterium*	↓	−0.288	0.072	0.120	0.000	0.000

*Verrucomicrobia*			↑	0.614	5.429	1.020	0.002	0.003
	*Verrucomicrobi*aceae		↑	0.629	5.396	1.009	0.006	0.008
		*Akkermansia*	↑	0.464	4.669	0.879	0.015	0.019
		*Prosthecobacter*	↑	0.511	0.133	0.022	0.000	0.000

*Cyanobacteria*			↓	−0.156	0.509	0.730	0.000	0.000
	*Aphanizomenonaceae*	*Dolichospermum*	↓	−1.152	0.009	0.391	0.000	0.000

a The results were obtained by Mann-Whitney U test performed on Statistical Package for the Social Sciences (SPSS) version 25.0, followed by the Benjamini and Hochberg false discovery rate (FDR) correction test for multiple comparisons.

b ↓, Significantly reduced in PD patients; ↑, significantly increased in PD patients.

c MD, mean difference between the logarithmic value of relative abundance in the PD and HC groups.

d Average relative abundance (as a percentage) of each taxon in the PD and HC groups.

e FDR-corrected *P* values with FDR < 0.05.

A total of 225 families in PDs and 217 in HCs were detected. The abundance of 16 families was significantly modified in PDs versus HCs. For instance, among the most relevant in PDs, *Verrucomicrobiaceae*, *Bifidobacteriaceae*, *Streptococcaceae*, and *Desulfohalobiaceae* were increased, while *Bacteroidaceae*, *Lachnospiraceae*, *Brevibacteriaceae*, and *Sphingobacteriaceae* families were reduced. Differences were also observed at the genus level (601 in PDs versus 563 in HCs). The microbiota of PD patients was characterized by significantly higher levels of several genera, such as *Akkermansia*, *Escherichia*, *Bifidobacterium*, *Streptococcus*, *Clostridium*, and *Serratia.* An increase in some genera, such as *Veillonella*, *Prosthecobacter*, *Enterobacter*, and *Slackia*, was also observed. In contrast, several genera were significantly reduced: *Bacteroides*, *Blautia*, *Lachnospira*, *Butyrivibrio*, *Roseburia*, *Pseudobutyrivibrio*, *Brevibacterium*, *Dolichospermum*, *Coprococcus*, and *Odoribacter*.

Interestingly, within *Firmicutes*, the major differences concerned *Lachnospiraceae*, whose different genera were significantly reduced in the PD group.

**FIG 3:**
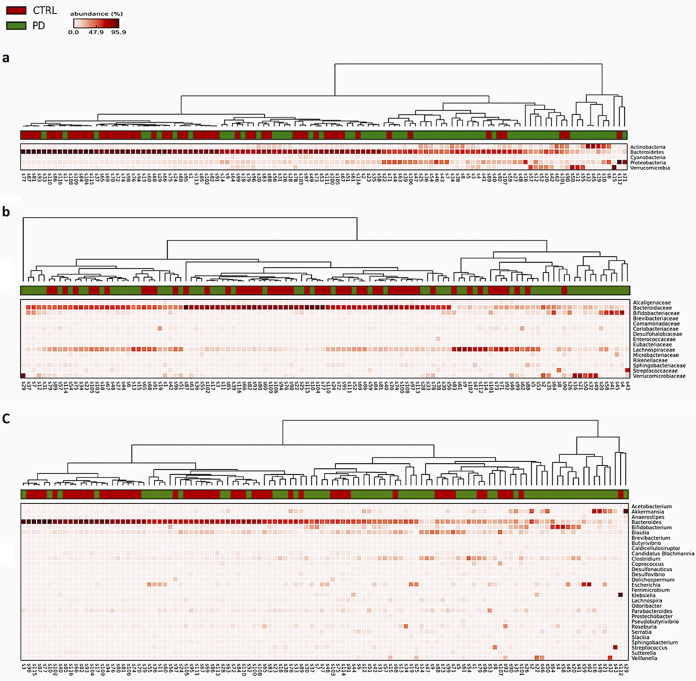
Heat maps of microbiota composition in PD cases and controls performed using the statistical analysis of metagenomic profiles (STAMP) software. The results were tested for statistical significance by the Mann-Whitney U test (Statistical Package for the Social Sciences Version [SPSS] 25.0) (60).

As for *Bacteroidetes*, the major reductions concerned *Bacteroidaceae* and members therein. An overview of abundant taxa of the gut microbiota composition from each PD and healthy subject is represented as a heat map using statistical analysis of metagenomic profiles (STAMP) software (Fig. 3).

The analysis for confounding factors was performed using the generalized linear model (GLM) that was adjusted for sex, age, body mass index (BMI), coffee consumption, and smoking. No differences were observed in terms of the Mediterranean diet among the different participants recruited for this study. GLM analysis confirmed only, in part, the results obtained by the univariate analysis, indicating that some confounding factors influenced the microbiota community of our samples. The statistically significant differences in the composition of the intestinal microbiota in the PD group compared to the HC group were, however, maintained at various taxonomic levels (Table 3). In particular, *Lachnospiraceae* was significantly depleted in PD patients. Accordingly, *Blautia*, *Butyrivibrio*, and *Coprococcus* were significantly reduced in the same group of subjects, while the only alteration in terms of richness was observed in the *Veillonella* genus. Concerning *Proteobacteria*, “*Candidatus* Blochmannia” was significantly reduced in PD patients. Also, *Brevibacteriaceae* and *Brevibacterium* belonging to *Actinobacteria*, as well as *Dolichospermum* belonging to *Cyanobacteria*, were significantly reduced. In our work, *Bacteroidetes* and *Verrucomicrobia* did not show any significant difference as opposed to the results of the univariate analysis.

**TABLE 3:** Statistically significant differences of gut microbiome in PD versus HC groups^*a*^

Phylum	Family	Genus	↓/↑^*b*^	MD^*c*^	*P* value	Bonferroni- adjusted *P* value^*d*^
*Firmicutes*	*Lachnospiraceae*		↓	−0.567	0.009	0.002
		*Blautia*	↓	−0.596	0.007	0.010
		*Butyrivibrio*	↓	−0.951	0.004	0.003
		*Coprococcus*	↓	−0.873	0.039	0.019
	*Veillonellaceae*	*Veillonella*	↑	1.556	0.002	0.000

*Proteobacteria*	*Enterobacteriaceae*	“*Candidatus* Blochmannia”	↓	−1.62	0.000	0.000
*Actinobacteria*	*Brevibacteriaceae*		↓	−0.640	0.001	0.000
		*Brevibacterium*	↓	−0.640	0.001	0.000

*Cyanobacteria*	*Aphanizomenonaceae*	*Dolichospermum*	↓	−1.364	0.001	0.000

a Analysis of covariance (ANCOVA) performed using generalized linear model (GLM) followed by Bonferroni correction for multiple comparisons in Statistical Package for the Social Sciences version (SPSS) 25.0 for Windows. The differences in microbiota composition between PD patients versus HCs were adjusted for sex, age, BMI, coffee consumption, and smoking status covariates.

b ↓, Significantly reduced in PD patients; ↑, significantly increased in PD patients.

c MD, mean difference between logarithmic value of relative abundance in the PD and HC groups.

d Bonferroni-corrected *P* values with *P* < 0.05.

### Evaluation of fecal metabolites reveals alterations in the gut metabolome in PD patients.

**FIG 4:**
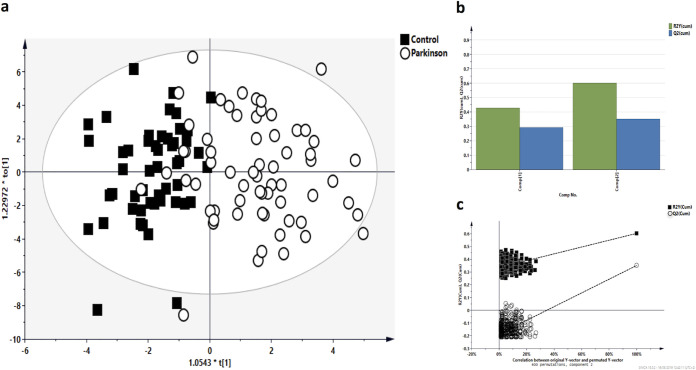
Metabolomics multivariate statistical analysis (MVA). (a) OPLS-DA score plots of PD patients versus control subjects. (b) Validation parameters. R2X and R2Y indicated the cumulative explained fraction of the variation of the X block and Y block for the extracted components. Q2 values indicated cumulative predicted fraction of the variation of the Y block for the extracted components. R2 and Q2 intercept values are indicative of a valid model. (c) The permutation test was evaluated on the corresponding partial least-square discriminant analysis (PLS-DA) model.

We performed a direct evaluation of fecal metabolites by gas chromatography-mass spectrometry (GC-MS). A total of 90 metabolites were identified that included organic compounds, lipids, amino acids, and vitamins. The results of the orthogonal partial least-square discriminant analysis (OPLS-DA) model obtained from the comparisons of PD and HC groups using multivariate statistical analysis (MVA) are shown in Fig. 4a. OPLS-DA model quality parameters (R2Y, 0.6; Q2, 0.36) and the respective permutation test (R2 intercept, 0.0, 0.335; Q2 intercept, 0.0, −0.187) are shown in Fig. 4b and c, displaying the statistical validity of the analysis and indicating distinct metabolic profiles in the two different groups (Fig. 4).

**FIG 5:**
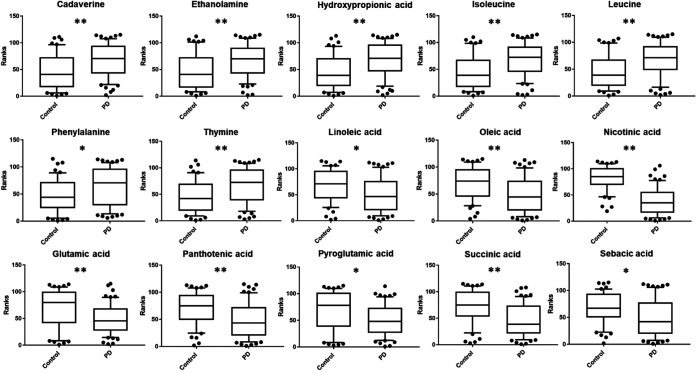
Statistically significant metabolites in fecal samples of PD patients versus control subjects comparison. Discriminant metabolites obtained with the MVA underwent a Mann-Whitney U test with Holm-Bonferroni correction to determine which metabolites were statistically significantly different. The resulted metabolites obtained are shown and expressed on the *y* axes of the graphs as ranks (data transformation in which numerical or ordinal values are replaced by their rank when the data are sorted). The levels of significance are indicated by asterisks as follows: *, *P* < 0.05; **, *P* < 0.01.

Both up- and downregulation of metabolites were observed in the PD group compared to the HC group. The PD fecal samples clearly showed higher levels of several metabolites, such as cadaverine, ethanolamine, hydroxypropionic acid, isoleucine and leucine, phenylalanine, and thymine. In contrast, linoleic acid, oleic acid, nicotinic acid, glutamic acid, pantothenic acid, pyroglutamic acid, succinic acid, and sebacic acid were significantly decreased (Fig. 5).

### Gut microbiota and fecal metabolite alterations are significantly related in PD patients.

We correlated the different patterns of changes observed in the microbiota composition with microbiota metabolites in PD and HC groups.

**FIG 6:**
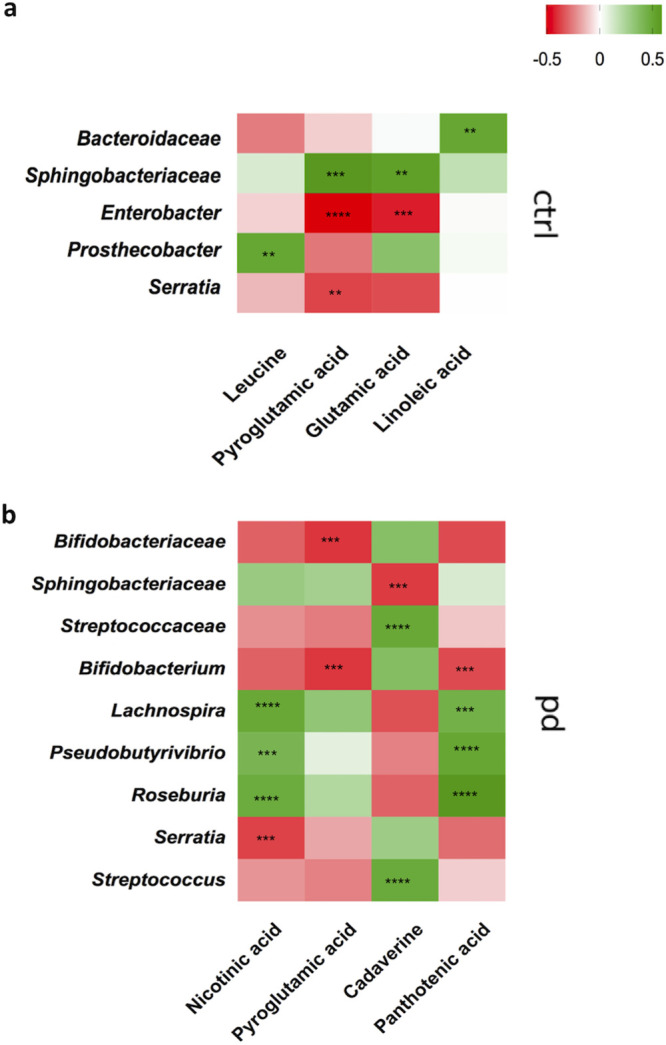
The heat maps represent Spearman correlation of the relative abundance of differential bacteria, selected by the linear discriminant analysis effect size (LEfSe) method followed by FDR correction test, and the concentrations of metabolites, selected by the MVA, underwent a Mann-Whitney U test with Holm-Bonferroni correction. (a) Heat map control (*n* = 51); (b) heat map PD (*n* = 64). The *r* values are represented by gradient colors, where red and green cells indicate positive and negative correlations, respectively. The asterisks indicate levels of significance as follows: *, *P* < 0.05; **, *P* < 0.01; ***, *P* < 0.001; ****, *P* < 0.0001.

The Spearman correlation analysis showed several significant associations of gut bacteria with metabolites in the two groups (Fig. 6). In PDs, *Lachnospira*, *Pseudobutyrivibrio*, and *Roseburia* genera showed strong positive correlations with nicotinic acid and pantothenic acid. Negative correlations were instead obtained between the *Serratia* genus and nicotinic acid, while *Bifidobacterium* correlated with pantothenic acid. *Streptococcaceae* and *Streptococcus* showed positive associations with cadaverine and, at the same time, a negative correlation with the *Sphingobacteriaceae* family. Negative correlations were also observed between the *Bifidobacteriaceae* family, and the related genus *Bifidobacterium*, with pyroglutamic acid. A positive correlation was obtained between this amino acid and the *Sphingobacteriaceae* family in the HC group. Conversely, negative correlations were observed with this amino acid and *Enterobacter* and *Serratia* genera. A similar trend was obtained between the *Sphingobacteriaceae* family and *Enterobacter* genus with glutamic acid. *Prosthecobacter* showed a positive correlation with leucine. Last, a positive correlation between *Bacteroidaceae* and linoleic acid was observed.

## DISCUSSION

The present study confirmed and extended previous studies by showing that the overall composition of gut bacterial microbiota in PD patients and HCs is significantly different. Moreover, evaluation of gut microbiota composition in relation to some confounding factors showed that some of these factors influenced the microbiota community, although the differences in the composition of the intestinal microbiota were maintained between PDs and HCs.

In recent years, the PD research community has given increasing attention to the alteration of gut microbiota because of its putative implication in the disease pathogenesis (27). Several reports on this topic have shown a different abundance of distinct bacterial taxa between PD patients and HCs. It has been postulated that this could be due to differences in the methodologies and patient enrollment criteria. This can also explain why data are only partially in agreement with each other (12–20).

The results presented herein show a distinctive profile of the gut microbial community in patients with PD compared to HCs. However, our results are in partial agreement with those previously produced by others. A possible explanation is that the statistical significance can be affected by the correction for confounders. As an example, in the case of *Firmicutes*, although univariate analysis showed a significant difference between patients and controls, after correction for different covariates (age, sex, BMI, coffee, and smoking), it did not match even with our data. However, in PD patients, the results showed a significant decrease in the abundance of taxa belonging to *Firmicutes*, particularly in *Lachnospiraceae* and key members therein, such as *Blautia*, *Coprococcus*, and *Butyrivibrio*. Our data are in agreement with other studies that showed a reduction of the *Lachnospiraceae* family and related genera in fecal samples from PD patients (12, 13, 18). Several members of the *Lachnospiraceae* family have progressively captured attention due to their ability to produce short-chain fatty acids (SCFAs) (28) (Table 4). Acetate, propionate, and butyrate are the primary SCFA molecules produced from gut bacteria fermentation and are endowed with anti-inflammatory properties. These metabolites appear to play an important role in orchestrating the function of the enteric nervous system and in promoting gastrointestinal integrity and motility (29). A reduction of SCFAs may also contribute to the development of gastrointestinal motility dysfunctions, thus highlighting the potential role of SCFA-producing bacteria in the pathogenesis of PD. We have investigated whether a reduction of the aforementioned SCFA producers correlates with the depletion of these metabolites in fecal samples from PDs. Although the levels of butyric acid, propionic acid, and acetic acid, in line with the microbiota profile relative to *Lachnospiraceae* members, were decreased in PD patients, no statistically significant variations were observed after FDR correction (*P* > 0.05). This finding is in contrast with the results by another research group that found a reduction in the fecal SCFAs (13). One possible explanation for this contrasting result may reside in the parallel increase we observed of *Veillonella*, *Akkermansia*, and *Clostridium* genera, which are known to produce acetate/propionate and butyrate. The enrichment of these bacteria may have caused a shift in the final levels of SCFAs, mirroring the balance between these taxa. Moreover, differences in the cohort studied, in terms of the number of participants (we considered a larger cohort) and other variables, including ethnic origins and host genetics, may also explain these discrepancies.

**TABLE 4:** PD-associated abundance of SCFA-producing bacteria^*a*^

Phylum	Family	Genus	↓/↑^*b*^	FDR- adjusted *P* value^*c*^	SCFA production
*Actinobacteria*			↑	0.003	Acetate
	*Bifidobacteriaceae*		↑	0.018	Acetate
		*Bifidobacterium*	↑	0.020	Acetate

*Bacteroidetes*			↓	0.000	Propionate
	*Bacteroidaceae*		↓	0.000	Propionate
		*Bacteroides*	↓	0.000	Propionate
	*Odoribacteriaceae*	*Odoribacter*	↓	0.002	Butyrate

*Firmicutes*	*Clostridiaceae*	*Clostridium*	↑	0.016	Butyrate
	*Lachnospiraceae* ^*d*^		↓	0.002	Butyrate
		*Blautia* ^*d*^	↓	0.002	Butyrate
		*Lachnospira*	↓	0.000	Butyrate
	*Veillonellaceae*	*Veillonella* ^*d*^	↑	0.000	Acetate/propionate

*Verrucomicrobia*			↑	0.003	Acetate/propionate
	*Verrucomicrobiaceae*		↑	0.008	Acetate/propionate
		*Akkermansia*	↑	0.019	Acetate/propionate

a Abundance of SCFA-producing bacteria in PD patients versus healthy controls using Mann-Whitney U test, followed by the Benjamini and Hochberg false discovery rate (FDR) correction test for multiple comparisons (FDR < 0.05).

b ↓, Significantly reduced in PD patients; ↑, significantly increased in PD patients.

c Bonferroni-corrected *P* values with *P* < 0.05.

d Abundance of SCFA-producing bacteria in PD patients versus controls adjusted for sex, age, and BMI covariates using analysis of covariance (ANCOVA) performed using a generalized linear model (GLM) followed by Bonferroni correction for multiple comparisons in Statistical Package for the Social Sciences Version (SPSS) 25.0 for Windows.

As mentioned earlier, we found an increase of *Veillonella* in the PD group. Our result extended, at the genus level, a previous report that showed an increase of the *Veillonellaceae* family in PD patients (14). In agreement and extending the results of another study (30) reporting a reduction of the *Brevibacteriaceae* family, we also found a decrease of the *Brevibacterium* genus.

Among the different changes reported, the reduction in “*Candidatus* Blochmannia” and *Dolichospermum* genera in PD patients is of particular interest. It should be noted that to date, none of the studies carried out on the microbiota in PDs showed changes in *Cyanobacteria*, to which the *Dolichospermum* genus belongs. Members of *Cyanobacteria* produce a series of neurotoxins that are implicated in the protein misfolding and aggregation phenomenon that is seen in PD and other neurodegenerative disorders (31). However, the role of *Dolichospermum* is not clear, and the decrease in the abundance of *Dolichospermum* in PD patients deserves further investigation concerning its potential pathophysiological role and the effects of this reduction.

Finally, an evaluation of the fecal metabolic profile was made, and it highlighted interesting differences between PD patients and HCs. In particular, our data from direct analysis of fecal metabolites revealed that the metabolism of several amino acids, such as phenylalanine, leucine, and isoleucine, was significantly increased in PD patients. A recent paper showed that the levels of phenylalanine were increased in the plasma of subjects with PD (32). Other work has reported that amino acid-fermenting bacteria could modulate the distribution of amino acids in the gastrointestinal tract (33, 34) and that altered amino acid concentrations may reveal changes in energy metabolism (35). Accordingly, the data reported in the present study indicate a significant depletion of energy metabolism in PD.

Notably, PD patients revealed a significant reduction of the glutamic acid derivative pyroglutamic acid, whereas glutamic acid is a neurotransmitter implicated in PD pathogenesis (36).

Although the results concerning the levels of glutamic acid associated with PD are rather discordant among studies, some studies have suggested that a reduction of glutamic acid, which is also a precursor of glutathione, may reflect an increase in oxidative stress in the disease progression (37).

In addition to modification in amino acid metabolism, our findings highlighted an alteration of lipid metabolism.

Interestingly, the PD group was characterized by a reduction of linoleic acid and oleic acid. In particular, linoleic acid is an omega-6 polyunsaturated fatty acid (PUFA), and it has been associated with protective effects. Previous studies have proposed that a reduction of PUFA in PD models may reflect an excess of oxidative stress (38). In line with our data, some authors reported that the serum of PD patients showed decreased levels of several long-chain PUFAs, including linoleic acid (39).

In addition to the above reported metabolic changes, we found a reduction of B vitamins, such as nicotinic acid (vitamin B3) and pantothenic acid (vitamin B5). Both these vitamins can be directly produced and secreted by commensal bacteria in the gut; they can also elicit anti-inflammatory and antioxidant activity and show protective effects against neurodegenerative mechanisms (40, 41). The reported decrease in the levels of both B3 and B5 vitamins strongly correlated with *Lachnospira*, *Pseudobutyrivibrio*, and *Roseburia* genera. Several genera of intestinal *Firmicutes* bacteria express crucial factors for vitamin B3 synthesis (42, 43), suggesting that these bacteria might affect the metabolism and bioavailability of these vitamins in the gut. Vitamin B5 is the primary precursor of coenzyme A, and its deficiency might be involved in the alteration of the citric acid cycle, causing defective energy levels, a finding shared by several neurodegenerative disorders, such as PD, Huntington’s disease, and Alzheimer’s disease (44).

Concerning vitamin B3, other investigations have found a chronic vitamin B3 deficit in PD patients (45).

A toxic effect is instead ascribed to the polyamine cadaverine (46), a product of bacterial and human cometabolism (47), which we found to be increased in PD patients. A recent study revealed that cadaverine is involved in the inhibition of intestinal motility in a mouse model (48). We reported that an increased level of cadaverine positively correlated with the *Streptococcaceae* family and the related genus *Streptococcus*, which are known to express cadaverine biosynthetic enzymes (49). An increase of cadaverine may also contribute to promoting a proinflammatory environment and motility dysfunctions of the gastrointestinal tract in PD.

Overall, our data highlight that microbiota modification correlated with numerous fecal metabolites, which is suggestive of the fact that PD is associated with gut dysregulation. Moreover, the present findings highlight that there is a mutualistic relationship between gut microbes and several bacterial metabolites that favor altered gut homeostasis.

In addition, we revealed alterations of several specific microbial taxa, including the reduction of the *Lachnospiraceae* family and genera therein (SCFA-producing bacteria), whose anti-inflammatory and protective role is well known within the organism.

Our study provides an overview of the complex alterations associated with PD. Since the interaction between gut microbiota and dopaminergic medication as well as anticholinergics has only recently been recognized (18, 50), more detailed investigations are needed and are in progress in order to establish the potential role played by gut bacteria on the therapeutic drugs in use (51) or, vice versa, a direct influence of the drugs themselves on microbiota modifications, as the drugs may hide the real microbiota composition in naive untreated PD patients. In addition, such studies might lead to the identification of novel candidate biomarkers for PD diagnosis and treatment and may provide a rationale for the development of new complementary therapeutic strategies for PD.

## MATERIALS AND METHODS

### Patients and samples.

The institutional review boards and human subject committees at the participating institutions approved the study (protocol PG/2017/17817). Written or verbal informed consent was obtained from all enrolled participants: 64 patients with diagnosed PD and 51 healthy controls.

Patient inclusion criteria were as follows: diagnosis of idiopathic PD according to the UK Brain Bank criteria, Hoehn and Yahr stage I to IV, age between 45 and 85 years, and stable doses of dopaminergic treatment for at least 4 weeks before enrollment. The exclusion criteria were as follows: atypical or secondary Parkinsonism; the use of probiotic or antibiotic supplements for the 3 months before enrollment; the presence of a primary gastrointestinal disease; the concomitant presence of internal medicine, neurological, or unstable psychiatric illness together with severe cognitive impairment. Patients were evaluated by the Movement Disorder Society-sponsored revision of the Unified Parkinson’s Disease Rating Scale (MDS-UPDRS) III and IV (motor part and motor fluctuations/dyskinesia) (52), and the following battery of clinical scales/questionnaires: nonmotor symptom scale (NMSS) (53), Scale for Outcome in Parkinson’s disease-autonomic (SCOPA-AUT) (53), and Cognitive Assessment Montreal (MoCA) (54). Patients were classified as either tremor dominant (TD), with postural instability and gait difficulty (rigid-akinetic) (PIGD), or as dyskinetic patients. Stool samples from each subject were collected at outpatient facilities of the AO Brotzu and AOU Cagliari hospitals (Cagliari, Sardinia, Italy) and delivered to the laboratory within 3 h. The control group was composed of healthy participants, selected among spouses and family members of study patients.

### Sample and library preparation and sequencing.

DNA extraction and purification were performed as previously described (46). In particular, DNA extraction from thawed fecal samples was performed using the QIAamp DNA stool minikit following the manufacturer’s instructions (Qiagen).

A total of 115 samples were sequenced using an Illumina MiSeq system. Sequences were assigned to operational taxonomic units (OTUs) using Quantitative Insights into Microbial Ecology (QIIME) (55). We performed a closed-reference OTU assignment using the “uclust” software (56) with a 97% sequence similarity threshold against Greengenes_13.8 97% OTU cluster (57) as a reference.

Barcoded amplicon libraries for sequencing on the Illumina MiSeq platform were generated using degenerate primers targeting the bacterial V3-V4 16S rRNA region with the Nextera index kit (Illumina), as previously described (46).

### Data and statistical analysis.

Analysis of the data generated on the Miseq system was carried out using the BaseSpace 16S Metagenomics app (Illumina). Operational taxonomic unit mapping to the Greengenes database (V.13.8) was performed using the QIIME platform (V.1.8.0). Alpha-diversity analysis (Shannon, Simpson, Fisher, Chao1, and abundance-based coverage estimator [ACE]), was performed on the Microbiome Analyst tool (58). For beta-diversity analysis, weighted and unweighted UniFrac and Bray-Curtis distances were calculated using Adonis in vegan R package (59).

The Mann-Whitney U test followed by FDR correction test for multiple comparisons was used to identify bacterial taxa that were statistically different among PD patients and controls.

Linear discriminant analysis effect size (LEfSE) (http://huttenhower.sph.harvard.edu/galaxy/) analysis was performed on a Galaxy computational tool to estimate the effect size of each differentially abundant feature. Results were then corrected by FDR correction test for multiple comparisons. Heat maps of gut microbiota composition were generated using STAMP software.

The GLM was implemented, followed by Bonferroni correction for multiple comparisons using Statistical Package for the Social Sciences version (SPSS) 25.0 for Windows (60), to test for confounding factors. Only bacteria that were found to be significant at the univariate level after FDR correction were considered. No normally distributed variables had been normalized using their logarithmic value before performing GLM. The differences in microbiota composition between cases and controls were adjusted for sex, age, BMI, coffee consumption, and smoking status covariates.

### Microbiota and metabolome analyses.

Fecal microbiota analysis was investigated as previously described (46). For metabolomics, frozen feces were mixed in methanol solution and sonicated. After centrifugation, the supernatants were dried and derivatized with methoxyamine dissolved in pyridine (Sigma-Aldrich, St. Louis, MO, USA). *N*-Methyl-*N*-(trimethylsilyl)-trifluoroacetamide (Sigma-Aldrich, St. Louis, MO, USA) was added, and the samples were resuspended in hexane and filtered. Then, 20 μl from each sample was used to create a pool for quality control.

For gas chromatography-mass spectrometry (GC-MS) analysis, 1 μl of the derivatized sample was injected splitless into a 7890A gas chromatography coupled with a 5975C network mass spectrometer (Agilent Technologies, Santa Clara, CA, USA) equipped with a fused silica capillary column. The gas flow rate through the column was 1 ml/min. Identification of metabolites was performed using the standard National Institute of Standards and Technology NIST 08 standard and Golm Metabolome Database (GMD) mass spectra libraries and by comparison with authentic standards. Data processing was performed using a pipeline in KNIME.

The multivariate statistical analysis, PLS-DA, and OPLS-DA were performed using SIMCA-P software (ver. 14.0; Umetrics, Sweden). GraphPad Prism software (version 7.01; GraphPad Software, CA, USA) was used to perform the Mann-Whitney U test with Holm-Bonferroni corrected *P* values and Spearman correlations between the microbiome and the metabolome.

### Data availability.

Sequencing data have been deposited in the European Nucleotide Archive (ENA) under the accession number PRJEB30401. Metadata have been deposited under the accession number PRJEB36138.
